# Editorial: Psychometrics in psychiatry 2022: aging psychiatry

**DOI:** 10.3389/fpsyt.2024.1460575

**Published:** 2024-07-26

**Authors:** Joyce Siette, Marianna Mazza

**Affiliations:** ^1^ The MARCS Institute for Brain, Behaviour and Development, Western Sydney University, Westmead, NSW, Australia; ^2^ Unit of Psychiatry, Fondazione Policlinico Universitario A. Gemelli IRCCS, Rome, Italy; ^3^ Department of Neurosciences, Università Cattolica del Sacro Cuore, Rome, Italy

**Keywords:** psychometrics, older adults, standardization, theory, psychiatry, history

The field of aging psychiatry is undergoing a significant transformation, propelled by the rigorous application of psychometric principles to better define and measure clinical constructs. This Editorial provides a comprehensive overview of the contributions made by four original papers within the “Psychometrics in Aging Psychiatry” series. These articles collectively describe the advancements and challenges in the measurement and assessment of psychiatric symptoms and cognitive functions in older adults, highlighting the importance of robust psychometric tools in clinical practice and dictate the need for continuous innovation in assessment methodologies.

Psychometrics, the scientific discipline concerned with the theory and technique of psychological measurement, traces its origins back to the late 19^th^ century (1). The foundations of psychometrics were laid in late 1880s at the University of Cambridge, where the first laboratory dedicated to this science was established. This pioneering effort marked the beginning of a systematic approach to measuring cognitive abilities, personality traits, and other psychological attributes (2). By the turn of the century and over time, up to the modern era, measurement approaches have evolved and perfected with the aim to construct reliable and valid tests, inspired by theoretical principles which could be applied to different clinical settings and research protocols (3).

In psychiatry, psychometrics has established a key role by providing reliable and valid tools for defining mental health conditions, ensuring accurate diagnosis, and guiding suitable treatments (4). Administering standardized tests to measure psychological functioning and describe personality characteristics of patients diagnosed with psychiatric diseases supports evidence-based assessment and contributes to tailored care and better evaluation of the efficacy of both psychological and pharmacological interventions (5).

The introduction of psychometric methods revolutionized the field, allowing for the quantification of complex psychological phenomena and facilitating large-scale epidemiological studies (6–8). In the context of aging psychiatry, psychometrics is particularly vital due to the multifaceted nature of psychiatric disorders in older adults, which often involve overlapping symptoms of cognitive decline and mood disturbances. In this context, psychometrics not only facilitates the identification of specific pathologies and favors differential diagnosis, but also permits to monitor functional consequences of diseases (9). By employing sophisticated measurement techniques, psychometrics can support and enhance our understanding and management of disorders, which consequentially is likely to improve patient outcomes.

Vaccarino et al. present a critical evaluation of the Quick Inventory of Depressive Symptomatology Self-Report (QIDS-SR) within populations suffering from neurodegenerative disorders (ND) compared to those with major depressive disorder (MDD). Applying Rasch Measurement Theory, the study confirms the QIDS-SR’s suitability for both cohorts, demonstrating unidimensionality and satisfactory category ordering. However, it also reveals gaps in item targeting, suggesting that the QIDS-SR may not effectively delineate levels of depressive severity in ND patients. These gaps suggest that while the QIDS-SR is effective for general depressive symptom screening, it requires refinement to capture the specific depressive conditions in ND. This paper reinforces the call for specialized tools to better serve the diagnostic needs of individuals with neurodegenerative conditions.

Samtani et al. provide a thorough review of social cognition assessments in individuals with neurocognitive impairments. They emphasize the critical role of social cognition in clinical decision-making and treatment planning. Their paper advocates for the adoption of validated, multicomponent and multidimensional measures that can accurately capture the social cognitive deficits in this population cohort. By establishing reliable tools, clinicians can better predict and monitor changes, ultimately improving patient care. This review urges the need for a standardized approach to social cognition assessment, which will be vital for comprehensive neuropsychiatric evaluations.

Yang et al. explore the factors influencing insight and treatment attitudes among hospitalized older patients with major depression. Their study identifies cognitive function, type of admission (e.g., involuntary), dependence on a caregiver, social functionality, and length of hospital stay as significant predictors. Their findings suggest that improving cognitive function and social support systems, alongside careful consideration of admission types, could enhance treatment outcomes. This research further describes the associations that exist between cognitive and social factors in managing depression in older hospitalized adults, highlighting areas for targeted interventions.

Saldivia et al. establish the relationships between life satisfaction, positive affect, depression, and anxiety among older adults in Chile. The study identifies multiple variables, including geographical area, sex, age, education level, household composition, partner status, employment status, caregiver status, economic satisfaction, chronic disease presence, medication use, and alcohol consumption as significant predictors. The research team found that satisfaction with health emerged as the most influential predictor for positive affect and depressive and anxiety symptoms. This research highlights the multifaceted nature of well-being in older adults, emphasizing the importance of addressing both positive and negative dimensions to improve mental health outcomes.

Our “Psychometrics in Aging Psychiatry” series provides new insights into the application of psychometric principles to aging populations. Together, these studies highlight the critical role of psychometrics in enhancing the assessment and treatment of psychiatric conditions in older adults. They also raise important questions about the need for tailored measurement tools that can accurately capture the complex and overlapping clinical symptoms experienced by this population. By advancing our understanding of these issues, this Research Topic contributes to the ongoing effort to improve mental health outcomes for older adults through more precise and effective psychometric assessments.

## Author contributions

JS: Writing – review & editing, Writing – original draft. MM: Writing – review & editing.
